# Allelic frequency of 12-*FGF4*RG and the association between the genotype with number of calcified intervertebral discs visible on radiographs in Coton de Tuléar and French Bulldog breeds

**DOI:** 10.1186/s12917-025-04573-7

**Published:** 2025-03-04

**Authors:** Vilma L. J. Reunanen, Tarja S. Jokinen, Liisa Lilja-Maula, Marjo K. Hytönen, Anu K. Lappalainen

**Affiliations:** 1 Department of Equine and Small Animal Medicine, University of Helsinki, Helsinki, Finland; 2 Department of Medical and Clinical Genetics, University of Helsinki, Helsinki, Finland; 3 Folkhälsan Research Center, Helsinki, Finland; 4 Department of Veterinary Biosciences, University of Helsinki, Helsinki, Finland

**Keywords:** *FGF4* retrogene_1_, Allele_2_, Dog_3_, Intervertebral disc disease_4_, Imaging_5_, Spine_6_, Grading_7_, Breeding_8_

## Abstract

**Background:**

Intervertebral disc disease (IVDD) is a major welfare issue in chondrodystrophic dogs. It is a consequence of chondroid metaplasia of the nucleus pulposus, leading to premature degeneration and calcification of the intervertebral discs (IVDs). Radiographic grading based on the number of calcified discs visible on radiograph (CDVR) between the ages of 24-48 months is an established method for selective breeding against IVDD in dogs. Premature IVD degeneration has a genetic background, and a *FGF4* retrogene insertion on chromosome 12 (12-*FGF4*RG) has been shown to be involved. The aim of this study was to determine the 12-*FGF4*RG allele frequency and genotype proportions, and the influence of the 12-*FGF4*RG genotype on number of CDVR in a study population of young adult Coton de Tuléars and French Bulldogs. In this combined prospective and retrospective analytical study, we investigated dogs radiographically screened at 24-48 months of age. The first dataset consisted of 12-*FGF4*RG genotyping results of 465 Coton de Tuléars and intervertebral disc calcification (IDC) grading results (no, mild, moderate, or severe) for 222 of them. The second dataset included 12-*FGF4*RG genotypes and IDC grading results (no or severe) of 81 French Bulldogs.

**Results:**

We observed 12-*FGF4*RG homozygous, heterozygous and wildtype individuals in both studied breeds. The 12-*FGF4*RG allele frequencies were also lower than previously reported in the studied breeds and Coton de Tuléars had lower allele frequency (0.35) than French Bulldogs (0.85). The distribution of IDC grading results were 59% no, 16% mild, 9% moderate and 16% severe in Coton de Tuléars and 59% no and 41% severe in French Bulldogs. In both breeds, every copy of the 12-*FGF4*RG allele significantly increased the risk for a higher number of CDVR, indicating incomplete dominance.

**Conclusions:**

Our results confirm the significant association between the 12-*FGF4*RG allele and the number of CDVR and IDC grade in two different chondrodystrophic breeds in age-controlled cohorts of young adult dogs. Our results also suggest that radiographic screening of CDVR and genetic testing of 12-*FGF4*RG could be used to breed against IVD degeneration predisposing to IVDD.

## Background

Intervertebral disc disease (IVDD) is a major welfare issue in many popular chondrodystrophic breeds such as Coton de Tuléar, Dachshund, and French Bulldog [1–3]. Clinical signs associated with IVDD may include severe pain, and/or neurological dysfunction such as paraplegia with loss of deep pain perception [4–8].

For many decades, it has been known that chondrodystrophic breeds are predisposed to premature intervertebral disc (IVD) degeneration due to a process called chondroid metaplasia, which usually results in intervertebral disc calcification (IDC) [6, 9–11]. There is evidence in Dachshunds that the maximum number of calcified discs visible on radiograph (CDVR) occurs at about 24-27 months of age, and the number of CDVR can subsequently decrease [12, 13]. CDVR correlates with the magnetic resonance imaging (MRI) IVD grading so, that the number of CDVR indicates the extent of overall IVD degeneration detected with MRI in young adult Dachshunds [14]. An increasing number of CDVR, increases the risk for clinical manifestation of an IVD degeneration [4, 6, 15, 16].

Premature IVD degeneration and the number of CDVR are highly heritable in Dachshunds, enabling breeding selection [17, 18]. Radiographic grading for the number of CDVR, especially at the age of 24-48 months, is already an established method and a useful tool for selective breeding against IDC, a risk factor for IVDD, in Dachshunds [13, 15, 16, 19]. In the Finnish Kennel Club, the grading scheme for CDVR has long been implemented also in the breeding programs of other chondrodystrophic breeds, including Coton de Tuléar and French Bulldog [20].

The term chondrodystrophy has previously been used in veterinary literature as a synonym for the short-limbed phenotype typical for these breeds. However, recent advances in canine genetics have revealed that short-limbed appearance is associated with two different retrogenes (RG), mostly with 18-*FGF4*RG (chondrodysplasia) and to a lesser extent with 12-*FGF4*RG (chondrodystrophy), with only 12-*FGF4*RG clearly predisposing to premature IVD degeneration [2, 21–24]. The 12-*FGF4*RG allele acts in a dominant manner and therefore even one copy of this retrogene increases the risk of early-onset IDC and IVDD [2, 21, 24]. In contrast, short-limbed breeds, such as West Highland White Terrier and Cairn Terrier, carrying only 18-*FGF4*RG [2, 25] have low clinical incidence of IDC and subsequently lower prevalence of IVDD [2, 22].

The short-limbed phenotype of dogs is very popular, and as a result, the 18-*FGF4*RG and 12-*FGF4*RG are common in numerous breeds [2, 21, 23, 25]. For instance, Dachshunds and French Bulldogs have extremely high allele frequency of 12-*FGF4*RG and high risk for IDC and IVDD [1–3, 21]. This creates a significant welfare issue, causing early morbidity and mortality, although differences in clinical presentation between the breeds exist [1, 5, 7, 8, 16]. Lower 12-*FGF4*RG allele frequency (0.58) has been observed in a small cohort of 12 Coton de Tuléars, while 18-*FGF4*RG seems to be almost fixed in this breed [2, 21]. French Bulldogs, in turn, are virtually free of the 18-*FGF4*RG allele [2, 25]. In breeds having both 12-*FGF4*RG and 18-*FGF4*RG alleles, moderate or low allele frequency of 12-*FGF4*RG offers the possibility to breed against risk for IDC and IVDD, while maintaining the short-limbed appearance [22]. However, the ethical perspective is also important to consider, when breeding extreme phenotypes.

Phenotypic variation and allele frequences are rather population-specific, and therefore, also the 12-*FGF4*RG allele frequencies and the proportion of dogs developing premature IVD degeneration and IVDD vary between breeds and countries [22]. For breeding purposes, it is important to conduct radiological screening for premature IVD degeneration at the age of 24-48 months, as aging can affect the number of CDVR [2, 14, 19]. According to the authors’ knowledge, to date, there are no studies exploring the association between number of CDVR and 12-*FGF4*RG status in an age-controlled population of young adult chondrodystrophic dogs other than Dachshunds [2, 14, 19].

The aims of this study were to determine the 12-*FGF4*RG allele frequency and genotype proportions and the influence of the 12-*FGF4*RG genotype on the number of CDVR in a study population of young adult Coton de Tuléars and French Bulldogs. We hypothesized that the allele frequency of 12-*FGF4*RG in Coton de Tuléars is lower than in French Bulldogs, and that the 12-*FGF4*RG genotype has a significant influence on the number of CDVR in both breeds.

## Results

### Coton de Tuléar

We obtained *12-FGF4*RG genotype results of 465 Coton de Tuléars. The allele frequency was 0.35. The 12-*FGF4*RG genotype proportions with sex distribution of these dogs are presented in Table 1.

**Table 1:** Proportions of 12-*FGF4*RG genotypes and sexes in different genotypes in Coton de Tuléar

**12-*FGF4*RG Genotype**	**All dogs**	**Females**	**Males**
Homozygous	61 (13%)	32 (52%)	29 (48%)
Heterozygous	199 (43%)	112 (56%)	87 (44%)
Wild-type	205 (44%)	112 (55%)	93 (45%)
Total	465 (100%)	256 (55%)	209 (45%)

12*-FGF4*RG* FGF4* retrogene insertion in chromosome 12

Altogether 222 of the 465 genotyped Coton de Tuléars had been radiographically screened for the number of CDVR at 24-48 months of age. The age at IDC grading, number of CDVR, and IDC grade distribution within the proportions of different 12-*FGF4*RG genotypes and sexes are presented in Table 2.

**Table 2:** Age at radiographic IDC grading, number of CDVR, and IDC grade distribution within proportions of different 12-*FGF4*RG genotypes and sexes in Coton de Tuléar dogs radiographed between the ages of 24-48 months

	**n**	**Age at IDC grading months: mean (SD) range**	**CDVR median (range)**	**n** **IDC 0**	**n** **IDC 1**	**n** **IDC 2**	**n** **IDC 3**
**12-*FGF4*RG genotype**							
Homozygous	41 (18%)	30.7 (4.8) 24 – 44	7 (0 – 18)	5 (12%)	4 (10%)	6 (15%)	26 (63%)
Heterozygous	100 (45%)	30.6 (7.0) 24 – 47	0 (0 – 5)	56 (56%)	22 (22%)	13 (13%)	9 (9%)
Wild-type	81 (36%)	29.5 (6.4) 24 – 48	0 (0 – 3)	71 (88%)	9 (11%)	1 (1%)	0 (0%)
Total	222 (100%)	30.4 (6.3) 24 – 48	0 (0 – 18)	132 (59%)	35 (16%)	20 (9%)	35 (16%)
**Sex**							
Female	117 (53%)	27.4 (6.0) 24 – 47	0 (0 – 16)	77 (66%)	15 (13%)	14 (12%)	11 (9%)
Male	105 (47%)	31.3 (6.5) 24 – 48	0 (0 – 18)	55 (52%)	20 (19%)	6 (6%)	24 (23%)

CDVR calcified discs visible on radiographs, IDC intervertebral disc calcification; IDC 0 = no, IDC 1 = mild, IDC 2 = moderate, IDC 3 = severe intervertebral disc calcification based on number of CDVR; 12-*FGF4*RG, *FGF4* retrogene insertion in chromosome 12

In Coton de Tuléar, males had a greater risk of having a higher number of CDVR (estimate =1.71, 95% CI 1.41-2.07, *P* <0.0001) and a higher IDC grade than females (OR =2.11, 95% CI 1.15-3.85, *P* =0.016). The age at screening had a significant effect on the number of CDVR (*P* =0.040), as the number of CDVR was somewhat reduced in dogs screened at an older age. However, the IDC grade in this breed was not affected by the age at screening (*P* =0.342).

The probability of having more CDVR or more severe IDC grade was higher in 12-*FGF4*RG homozygous dogs than in heterozygous dogs or wild-type dogs (*P* <0.0001). In addition, the probability of having more CDVR or more severe IDC grade was higher in heterozygous dogs than in wild-type dogs. The respective ORs, estimates, 95% CIs, and *P* -values are presented in Table 3.

**Table 3:** Effect of different 12-*FGF4*RG genotypes on number of CDVR and IDC grade 3 as Poisson regression estimates for number of CDVR and cumulative logit-model odds ratios for probability for having IDC grade 3 in Coton de Tuléar

**Variable**	**12-*FGF4*RG**	**Estimate**	**Lower CI**	**Upper CI**	***P***-value
	homozygous vs. heterozygous	4.88	3.99	5.96	<0.0001
Estimates for higher number of CDVR	homozygous vs. wild-type	36.67	21.82	61.63	<0.0001
	heterozygous vs. wild-type	7.52	4.42	12.81	<0.0001
	**12-*FGF4*RG**	**OR**	**Lower CI**	**Upper CI**	***P*-value**
	homozygous vs. heterozygous	15.34	6.78	34.7	<0.0001
Odds ratios for probability of IDC grade 3	homozygous vs. wild-type	92.45	33.51	255	<0.0001
	heterozygous vs. wild-type	6.02	2.76	13.12	<0.0001

12*-FGF4*RG, *FGF4* retrogene insertion in chromosome 12; CDVR calcified discs visible on radiographs; IDC grade 3 severe intervertebral disc calcification based on number of CDVR; OR odds ratio; *CI* 95% confidence interval

### French Bulldog

We obtained a total of 81 French Bulldogs´ CDVR screening results of IDC grades 0 or 3 and their 12-*FGF4*RG genotypes. The allele frequency was 0.85. The different 12-*FGF4*RG genotype proportions with sex distribution are presented in Table 4.

**Table 4:** Proportions of 12-*FGF4*RG genotypes and sexes in different genotypes in French Bulldogs with IDC grade 0 or 3

**12-*FGF4*RG Genotype**	**All dogs**	**Females**	**Males**
Homozygous	60 (74%)	34 (57%)	26 (43%)
Heterozygous	17 (21%)	11 (65%)	6 (35%)
Wild-type	4 (5%)	4 (100%)	0 (0%)
Total	81 (100%)	49 (60%)	32 (40%)

12*-FGF4*RG* FGF4* retrogene insertion in chromosome 12; IDC intervertebral disc calcification; IDC 0* =* no, IDC 3 = severe intervertebral disc calcification based on number of CDVR

The age at IDC grading, the number of CDVR, and the IDC grade distribution within the proportions of different genotypes and sexes are presented in Table 5.

**Table 5:** Age at radiographic IDC grading, range for number of CDVR, and IDC grade distribution and proportions within different 12-*FGF4*RG genotypes and sexes in French Bulldogs with IDC grade 0 or grade 3

	**n**	**Age at IDC grading months: mean (SD) range**	**CDVR range**	**n IDC0**	**n IDC3**
**12-*FGF4*RG genotype**					
Homozygous	60 (74%)	27.1 (6.9) 27 – 42	0 –10	31(52%)	29 (48%)
Heterozygous	17 (21%)	27.5 (4.7) 24 – 43	0 – 5	13 (76%)	4 (24%)
Wild-type	4 (5%)	32 (4.9) 24 – 49	0	4 (100%)	0 (0%)
Total	81 (100%)	27.4 (5.0) 24 – 49	0 – 10	48 (59%)	33 (41%)
**Sex**					
Female	49 (60%)	27.2 (5.4) 24 – 49	0 – 10	34 (69%)	15 (31%)
Male	32 (40%)	27.8 (4.4) 24 – 46	0 – 10	14 (44%)	18 (56%)

CDVR calcified discs visible on radiographs; IDC intervertebral disc calcification; IDC 0 = no, IDC 3 = severe intervertebral disc calcification based on number of CDVR; 12*-FGF4*RG* FGF4* retrogene insertion in chromosome 12

A trend for a sex difference was detected in the French Bulldog, as males seemed to have a higher probability of IDC grade 3 than females (OR =2.34, 95% CI 0.90-6.07), yet this was not statistically significant (*P* =0.082). Age at screening had no significant impact on IDC grade (0 or 3).

The different 12-*FGF4*RG genotypes (homozygous, heterozygous, and wild-type) did not alone have a significant effect on IDC grade (0 or 3). However, when heterozygous and wild-type dogs were combined and compared with the 12-*FGF4*RG homozygous dogs, the latter had a significantly higher probability of having IDC grade 3 than IDC grade 0 (OR =3.4, 95% CI 1.03-11.51, *P* =0.045).

## Discussion

In this study, we demonstrate the clear influence of the 12-*FGF4*RG genotype on the number of CDVR and IDC grade in a study population of young adult Coton de Tuléars and French Bulldogs. We observed diversity in 12-*FGF4*RG genotypes and slightly different 12-*FGF4*RG allele frequencies in Coton de Tuléars and French Bulldogs than have previously been reported [2, 21]. Our data showed that every copy of 12-*FGF4*RG clearly increases the risk for a higher number of CDVR and higher IDC grade.

Our study is the first to explore the association between number of CDVR and 12-*FGF4*RG status in an age-controlled population of young adult dogs. Earlier studies have investigated breeds where the 12-*FGF4*RG is almost fixed [14, 19]. In addition, one study explored the connection between 12-*FGF4*RG status and presence or absence of CDVR in surgically treated IVDD cases of any age [2]. Another study in Nova Scotia Duck Tolling Retrievers detected an association of 12-*FGF4*RG allele and calcified IVDs on computed tomography images, yet did not control for age [24]. Especially, the breeding bitches have to be selected as young adults. In addition, IVD degeneration is also a part of aging process in all dogs [10, 11]. Therefore, to selectively breed against premature IVD degeneration to reduce the risk of IVDD, it is important to be able to distinguish the individuals with early-onset IVD degeneration from unaffected individuals.

The number of CDVR has been found to be associated with more severe overall IVD degeneration even within 12-*FGF4*RG homozygous Dachshunds [14] and to increase the risk of IVDD [15, 16, 19, 26]. There is also evidence for an association between 12-*FGF4*RG allele and IVDD [2, 21]. As IVDD is one of the most common neurological diseases in dogs, its reduction would significantly increase canine welfare, especially in chondrodystrophic breeds [1–3, 19, 27]. In our study, approximately 90% of wild-type Coton de Tuléars had no CDVR and none of them had IDC grade 3, and the four wild-type French Bulldogs were also free of CDVR. Also, more than half of heterozygous dogs in both breeds were free of CDVR, while most 12-*FGF4*RG homozygous Coton de Tuléars had IDC grade 3 (severe). These findings support previous results of even one 12-*FGF4*RG allele increasing the risk for having CDVR [2, 21] or IVD calcifications detected in computed tomography images [24]. Our results also suggest that the allele shows incomplete dominance, as homozygosity of the 12-*FGF4*RG allele increases the risk for a higher number of CDVR and more severe IDC grade. This finding is clinically important as well as impacting the planning of breeding schemes.

Premature IVD degeneration is multifactorial, with a significant genetic predisposition and high heritability in dogs and humans [2, 15, 17, 18, 22, 28–31]. Besides environmental factors, genetic factors other than 12-*FGF4*RG might also affect premature IVD degeneration and subsequently IVDD [2, 22, 32]. These factors could explain why approximately 12% of wild-type Coton de Tuléars representing both sexes had at least one CDVR. Our result is comparable with previous evidence of wild-type dogs having calcified IVD and IVDD [2]. Furthermore, many 12-*FGF4*RG homozygous dogs in both breeds were free of CDVR. This could partly be explained by rather low sensitivity of radiography to detect all calcified IVDs [6, 33]. However, the differences in clinical presentation, age of onset, and prognosis of IVDD between breeds having almost fixed 12-*FGF4*RG also support the theory of other contributing factors; for example, French Bulldogs are generally affected at a younger age, have more severe neurological dysfunction, and have less favourable outcome than Dachshunds [2, 3, 5, 8, 34, 35].

Our study included two different chondrodystrophic breeds that were chosen based on the expected 12-*FGF4*RG allele frequencies [2, 21]. In both breeds, we detected even more diversity in genotype frequencies than we expected. In contrast to a previous study [2], the wild-type genotype was most common in Coton de Tuléars (44%) and only a minority were homozygous for 12-*FGF4*RG allele (13%). Altogether, 21% of French Bulldogs were heterozygous for 12-*FGF4*RG, and we even found four wild-type French Bulldogs. In other words, our 12-*FGF4*RG allele frequencies of 0.35 for Coton de Tuléars and 0.85 for French Bulldogs were lower than the previously reported 0.58-0.92 for Coton de Tuléars and 0.94-0.97 for French Bulldogs [2, 21]. Differences between study populations and our much larger study cohort than in earlier studies might explain the difference in Coton de Tuléars. For French Bulldogs, our inclusion criterion of radiographic grading result of 0 or ≥ 5 CDVR (IDC grades 0 and 3) explains the lower allele frequency, and we were able to show that some variation exists.

Contrary to a study on Dachshunds where females had a higher number of CDVR [18] and studies in which no sex predilection was detected in Dachshunds [14, 36, 37], we observed that males in both studied breeds had a greater risk for a higher number of CDVR and more severe IDC grade than females. However, this risk was a trend in French Bulldogs and statistically significant only in Coton de Tuléars. In many studies, concerning clinical IVDD in different breeds, males have been slightly overrepresented [1, 2, 5, 38–40]. Interestingly, a recent human study using magnetic resonance imaging reported a significantly increased risk of IVD degeneration in young adult men than women of a similar age [41]. The reason for this is not known, but physiological explanations have been proposed in humans [41]. In the current study, the highest numbers of CDVR were found in Coton de Tuléar males. This might also be clinically important by increasing the risk for IVDD as the number of CDVR rises [15, 16, 26].

Although we investigated dogs in the rather narrow age range of 24-48 months, we observed that older imaging age led to a significantly lower number of CDVR in Coton de Tuléars. One explanation can be the disappearance of CDVR from the intervertebral space by dissolution or herniation, which has been previously demonstrated in Dachshunds and French Bulldogs even at an early age [2, 5, 8, 12, 13]. This phenomenon is important to keep in mind when evaluating the risk for IVDD based on the number of CDVR. In our study, the imaging age did not have an effect on IDC grade. However, the explanation in most probably a difference in statistical power between continuous and categorical responses.

Our results are in line with previous studies in which radiographic screening for CDVR and 12-*FGF4*RG variant genotyping have been demonstrated to be feasible preventative measures against premature IVD degeneration and reduce the risk of IVDD [2, 13–16, 19]. We acquired knowledge about two populations with different genotype and allele frequencies, and this information will likely allow more efficient breeding schemes to be developed. In our study population, Coton de Tuléars had a moderately low 12-*FGF4*RG allele frequency, which probably enables the eradication of the 12-*FGF4*RG allele with time. This approach is recommended since we now know that the 12-*FGF4*RG allele predisposes the dogs to more severe IDC grades. Based on our findings, 12-*FGF4*RG genotyping and selecting of wild-type dogs to the breeding population are the primary methods for improving breeding schemes in this breed. In contrast, French Bulldogs’ 12-*FGF4*RG allele frequency is very high, but they have phenotypic variation in IDC grades. We suggest that this enables phenotypic selection based on radiographic screening for CDVR and excluding individuals with IDC grade 3 from breeding. Genotyping especially IDC grade 0 dogs to identify heterozygous and wild-type dogs in addition to radiographic screening could intensify the breeding selection also in French Bulldogs. These methods could also be applicable to breeding selection for reducing the risk for IDC and IVDD in other chondrodystrophic breeds if they have diversity in 12-*FGF4*RG allele or variation within IDC grades. However, the efficiency of the phenotypic breeding selection is clearly lower than selection based on genetic testing. Utilization of estimated breeding values could intensify the breeding selection. Further studies on the prevalence of 12-*FGF4*RG and its association with premature IVD degeneration in larger populations and different chondrodystrophic breeds will help to develop even better breeding schemes. In addition, studies about the residual variability of CDVR and IDC grade and the influence of the different tools for selective breeding against the risk of IVD degeneration and IVDD are warranted. If sufficient variation does not exist in a breed, a large-scale crossbreeding with 12-*FGF4*RG wild-type dogs is the only viable option.

This study has some limitations that should be addressed. In French Bulldogs, the small proportion of heterozygous and wild-type dogs caused imprecision in estimation of the effects related to the 12-*FGF4*RG genotype. Due to our dichotomous inclusion criteria, the calculated allele frequency cannot be generalized to the entire French Bulldog population even in Finland, but nevertheless shows that wild-type individuals can be found.

## Conclusion

Our results confirm the significant association of the 12-*FGF4*RG allele with the number of CDVR and IDC grade in two different chondrodystrophic breeds (Coton de Tuléar and French Bulldog) in age-controlled cohorts of young adult dogs. Our results will help to develop more effective breeding schemes to resist premature IVD degeneration and subsequent IVDD – a major welfare problem for chondrodystrophic breeds.

## Methods

This study was approved by the Research Ethics Committee on Animal Research of the University of Helsinki (statement 5/2023).

### Recruitment and data collection

This analytical study consisted of prospective and retrospective data collection. The material was collected in collaboration with the Finnish Coton de Tuléar and the Finnish French Bulldog breed clubs. The study protocol consisted of collecting 12-*FGF4*RG genotype results and grading results of CDVR of privately owned dogs between January 2017 and October 2022. The research project was advertised in breed magazines, breed clubs’ web pages, and the social media. Owners willing to participate in the research project filled in an informed consent form and sent their dogs’ CDVR grading results and 12-*FGF4*RG genotype results to the breed clubs, which then recorded the data.

The 12-*FGF4*RG genotyping was conducted in commercial laboratories (Laboklin, Bad Kissingen (Germany); University of California -Davis Veterinary Genetics Laboratory (US); Gensol animal diagnostics, Clayton (US); Dr. van Haeringen Laboratorium B.V., Wageningen (Netherlands)) using buccal swabs, and the grading for CDVR was performed as part of the Finnish Kennel Club routine IVDD screening protocol for chondrodystrophic breeds [20, 42]. This IVD degeneration screening protocol, which consists of four IVD calcification classes (IDC grades), is used internationally [16, 17]. The IDC grade is calculated based on the number of CDVR (Table 6.).

**Table 6:** Grading of intervertebral disc calcification (IDC) used internationally

	**IDC grade**			
	IDC 0	IDC 1	IDC 2	IDC 3
Number of CDVR	0	1 – 2	3 – 4	≥ 5

CDVR calcified discs visible on radiographs; IDC grade intervertebral disc calcification grade (Jensen and Christensen, 2000; Lappalainen et al., 2014); IDC 0 no, IDC 1 mild, IDC 2 moderate, IDC 3 severe intervertebral disc calcification based on number of CDVR

### Coton de Tuléar

We obtained the 12-*FGF4*RG genotype results from the breed club database to investigate the allele frequency in Coton de Tuléars. In addition, we used IDC grading results from the dogs radiographed between the ages of 24-48 months to evaluate the association between the number of CDVR/IDC grade and 12-*FGF4*RG genotype.

### French Bulldog

For French Bulldogs, we included only the dogs with a grading result of 0 or ≥ 5 CDVR (IDC grades 0 and 3) radiographed at 24-48 months of age. Thereafter, 12-*FGF4*RG genotyping was performed on these dogs. In French Bulldogs, the 12-*FGF4*RG frequency is known to be very high, and by including only dogs with IDC 0 and IDC 3 grades we wanted to ensure distinct phenotypic classification to increase the possibility of finding wild-type alleles to demonstrate more clearly the association between 12-*FGF4*RG allele and IDC grade.

### Statistical analyses

We calculated frequencies and percentages for demographic variables, the number of CDVR, IDC grade, and 12-*FGF4*RG allele frequency for both breeds separately.

To investigate the association between the number of CDVR and 12-*FGF4*RG genotype, sex, and age for Coton de Tuléar, Poisson regression analyses were used, where all 12-*FGF4*RG genotypes (12-*FGF4*RG homozygous, heterozygous, wild-type) were modeled. Genotype, sex, and age were included in the model as explanatory variables. For Coton de Tuléar, the effects of 12-*FGF4*RG genotype, age, and sex on IDC grade (0, 1, 2, or 3) were analyzed using a cumulative logit model with IDC grade as the response and 12-*FGF4*RG genotype and sex as fixed effects. Age was used as a covariate.

For the French Bulldog, the effects of 12-*FGF4*RG genotype, age, and sex on IDC grade (0 or 3) were analyzed using logistic regression model in two different ways, as their genetic data had a low number of observations in the wild-type genotype. Therefore, first the effects of all separate genotypes (12-*FGF4*RG homozygous, heterozygous, wild-type) and sex on IDC grades were analyzed applying Firth’s penalized likelihood in the model to account for the low cell frequencies. Then, a similar model was constructed in which heterozygous and wild-type genotypes were combined and examined against the 12-*FGF4*RG homozygous genotype. The 12-*FGF4*RG genotype and sex were used as fixed terms and age was used as a covariate.

To quantify potential associations, odds ratios (ORs) or estimates with 95% confidence intervals (CIs) were calculated from all models for both breeds. The models were constructed to evaluate the probability of higher IDC grade or higher number of CDVR.

All statistical analyses were selected and completed by a biostatistician using SAS® System for Windows, version 9.4 (SAS Institute Inc., Cary, NC, USA). *P*-values <0.05 were considered significant.

## Data Availability

The data will not be made openly available to third parties or outside the original research team for patient confidentiality reasons.

## Abbreviations

CDVR: Calcified discs visible on radiographs
IDC: Intervertebral disc calcification
IVD: Intervertebral disc
IVDD: Intervertebral disc disease
MRI: Magnetic resonance imaging
12-*FGF4*RG: *FGF4* retrogene insertion on chromosome 12, chondrodystrophy
18-*FGF4*RG: *FGF4* retrogene insertion on chromosome 18, chondrodysplasia
